# Multi-allelic phenotyping – A systematic approach for the simultaneous analysis of multiple induced mutations^☆^

**DOI:** 10.1016/j.ymeth.2013.04.013

**Published:** 2013-08-15

**Authors:** Christopher M. Dooley, Catherine Scahill, Fruzsina Fényes, Ross N.W. Kettleborough, Derek L. Stemple, Elisabeth M. Busch-Nentwich

**Affiliations:** Wellcome Trust Sanger Institute, Wellcome Trust Genome Campus, Hinxton, Cambridge CB10 1SA, UK

**Keywords:** Zebrafish, Knockout, Screen, Model organism, Phenomics, Cryopreservation

## Abstract

The zebrafish mutation project (ZMP) aims to generate a loss of function allele for every protein-coding gene, but importantly to also characterise the phenotypes of these alleles during the first five days of development. Such a large-scale screen requires a systematic approach both to identifying phenotypes, and also to linking those phenotypes to specific mutations. This phenotyping pipeline simultaneously assesses the consequences of multiple alleles in a two-step process. First, mutations that do not produce a visible phenotype during the first five days of development are identified, while a second round of phenotyping focuses on detailed analysis of those alleles that are suspected to cause a phenotype. Allele-specific PCR single nucleotide polymorphism (SNP) assays are used to genotype F2 parents and individual F3 fry for mutations known to be present in the F1 founder. With this method specific phenotypes can be linked to induced mutations. In addition a method is described for cryopreserving sperm samples of mutagenised males and their subsequent use for *in vitro* fertilisation to generate F2 families for phenotyping. Ultimately this approach will lead to the functional annotation of the zebrafish genome, which will deepen our understanding of gene function in development and disease.

## 1 Introduction

Whole genome sequence is now available for humans as well as a number of other vertebrate species. While this has provided detailed information about the position and sequence of protein coding genes, the functions of these genes and their roles in development and disease remain largely unknown. Loss of function analysis can be either an appealing starting point or an integral component in the endeavour to elucidate gene function.

Forward genetic screens [1–6] have typically taken advantage of mutagenic chemicals such as EMS (Ethyl methanesulfonate) and ENU (*N*-ethyl-*N*-nitrosourea) to produce rich collections of phenotypes. Identification of the underlying mutations responsible for these phenotypes still poses significant challenges even today [7].

**Fig. 1:**
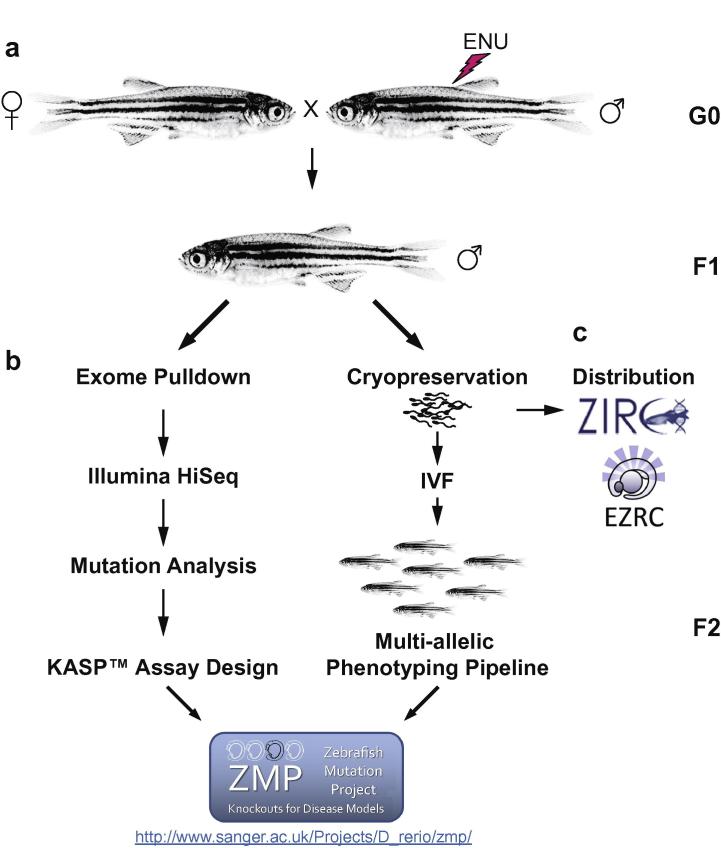
Mutation detection overview. Male TLF zebrafish (G0) are treated with ENU and then out-crossed (a) to produce F1 families heterozygously carrying induced mutations. F1 fish (b) are raised to an age of about 12 months and sperm is collected from the fish. Each individual is then sacrificed and tissue samples of the body are taken. The tails are used for genomic DNA preparation while the rest of the body is preserved for archival purposes. Genomic DNA is then subjected to exome pulldown (b), sequenced via Illumina paired end HiSeq and analysed to detect induced mutations. KASP™ assays are designed for each allele and then all information is made available on the ZMP website as the project proceeds. The cryopreserved sperm is made available to ZIRC and EZRC where alleles can be ordered. (c) An aliquot of sperm from each F1 male is retained at the Wellcome Trust Sanger Institute and used for the IVF of F2 families which are placed into the ZMP multi-allelic phenotyping pipeline. Phenotypic descriptions are published on the ZMP website.

The completed sequence of the zebrafish (*Danio rerio*) genome and its detailed annotation has yielded the sequence of more than 26,000 zebrafish protein coding genes [8,9]. Previously we have shown that it is possible to amplify specific exons across a large library of ENU mutagenised individuals and sequence these amplicons in order to identify mutations of interest, but these approaches are limited by the need for PCR amplification [10]. Alternatively, specific genomic regions can be enriched by hybridising fragmented DNA to selected regions of interest [11]. This has been used successfully to enrich genomic DNA sequencing libraries for whole exome analyses in human [12] and mouse [13]. To apply this technique in zebrafish, Agilent SureSelect™ RNA baits were designed to enrich for all protein coding exons identified in the Zv8 and Zv9 zebrafish genome assemblies [14]. Combined with Illumina sequencing and single nucleotide variant (SNV) calling [15–17], this method reliably identifies point mutations in thousands of individual fish thus taking advantage of the high mutagenic load ENU creates [18] (Fig. 1b). Sperm from the sequenced F1 males (Fig. 1a) is archived by cryopreservation and therefore desired alleles can be prioritised for *in vitro* fertilisation and revived into F2 families (Fig. 1b). Competitive allele-specific PCR SNP genotyping assays (KASP™, KBioscience) are designed [19] for the identified mutations and published on the ZMP website (Fig. 1b). The phenotypic consequences of all non-synonymous alleles contained in the preserved founder are addressed in a multi-allelic phenotyping pipeline, creating a crossroads of both forward and reverse genetics [20].

## 2 Cryopreservation

Cryopreserving sperm of F1 individuals into multiple aliquots as they are being sequenced allows for permanent archiving of identified alleles, distribution and selection of families to phenotype based on allele composition (Fig. 1c). Several zebrafish sperm cryopreservation protocols have been described previously [21–23]. They are based on either dissection of testis, thereby sacrificing the males, or retrieval of sperm by abdominal massage and use different types of cryoprotectants yielding different numbers of samples per male. The method described here is a combination and modification of protocols mentioned above. It uses N,N-dimethylacetamide (DMA) in buffered sperm-motility inhibiting solution (BSMIS) as the cryoprotectant and yields eight samples per male. As males are not sacrificed during this procedure they can be re-used after a rest period.

Males that are to be used for cryopreservation should be between 6 and 12 months old. It is important to keep them at a low stock density and to feed them well to generate relatively large fish. Sperm quantity can be increased significantly by separating males from females at least a week in advance of cryopreservation. Sperm quantity and quality do not deteriorate by prolonged sex separation. A well-fed male that has been separated from females can easily produce 4 μl of high density sperm.

**Fig. 2:**
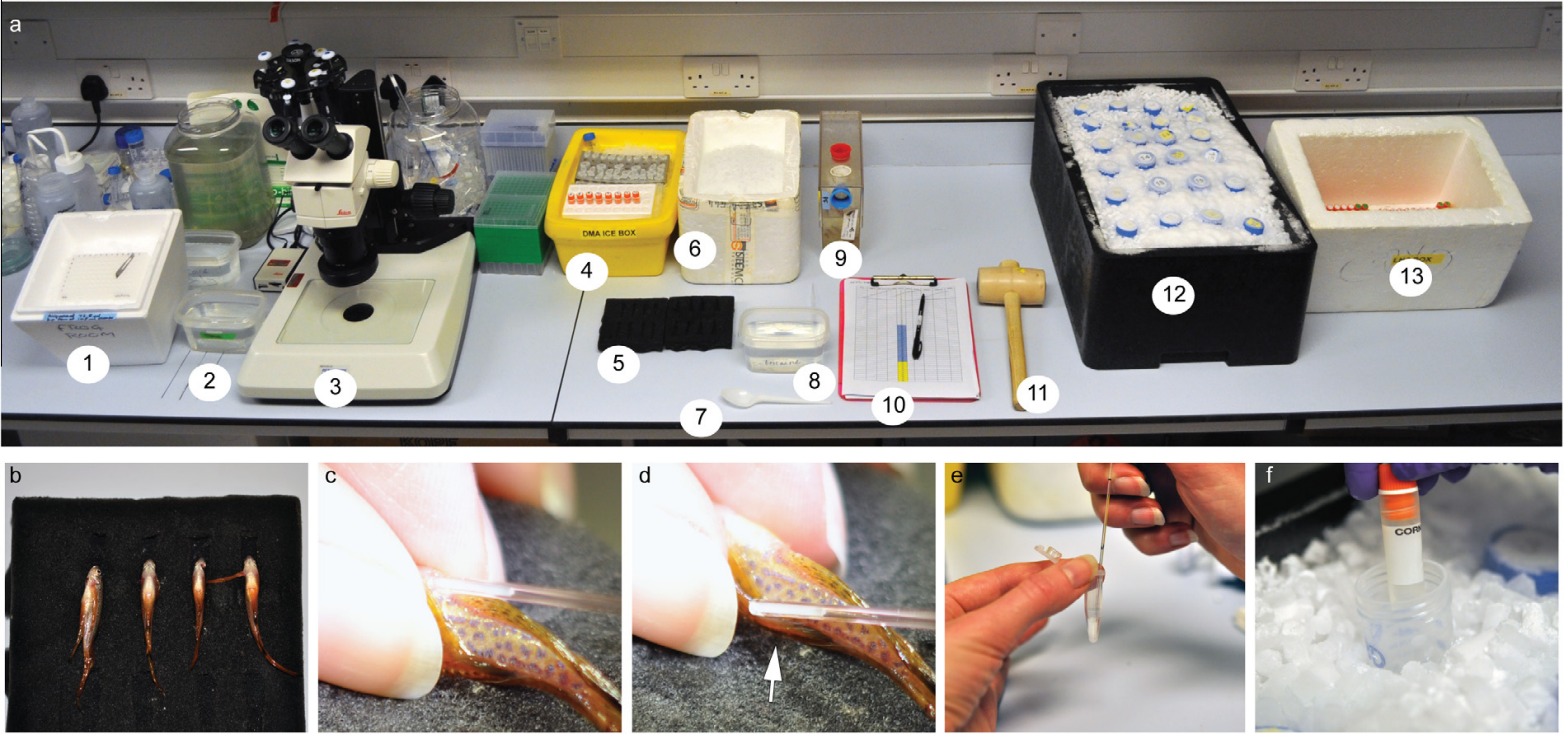
Cryopreservation of zebrafish sperm. From left to right the cryopreservation station comprises (1) dry ice block with deep well block for F1 tissue samples, (2) tricaine for culling, (3) LM80 dissecting microscope fitted with ring light, 1000 and 200 μl pipettes, bin and pipette tips behind, (4) wet ice box with tube rack for cryoprotectant aliquots and cryovials, (5) sponge with slits to hold anaesthetised fish, (6) wet ice box for cryovial box, (7) plastic spoon to scoop fish out of tricaine, (8) tricaine for anaesthesia, (9) fish tank holding males, (10) recording sheet, (11) mallet to drive Falcon tubes into dry ice, (12) dry ice/ethanol box with Falcon tubes, (13) styrofoam box containing 3 cm of LN_2_ and cryoboxes. Not depicted are: timer, tissues and cotton buds to dry off fish, suction tube and capillaries. (a) Males are placed in sponge holder ventral side up. (b) Carefully dried males are squeezed and sperm is collected in 10 μl capillary. (c) Good quality sperm is visible inside the capillary (white arrow). (d) Sperm is expelled into cryoprotectant while holding tube such that the solution is not warmed between the fingers. (e) Aliquots are pipetted into cryovials and immediately dropped into Falcon tubes which are capped and then hammered into the dry ice (f).

Depending on the number of samples to be frozen, ideally two or three people should work together on this protocol. One person opens cryovials (Corning Product #430659), retrieves the sperm and aliquots it into the cryovials while a second person anaesthetises males, records data and closes and transfers cryovials into the 50 ml Falcon tubes on dry ice and later into liquid nitrogen (LN_2_). For maximal throughput a third person can take over opening and closing of Falcon tubes and moving of cryovials into LN_2_, while the second person focuses on fish anaesthesia, data records and transfer of vials into Falcon tubes. If tissue samples for DNA isolation are also desired, a fourth person sacrifices the squeezed male fish by over-anaesthesia, takes tissue samples such as the tail fin and a section of the trunk and places those into a 96 deep well block on dry ice. It is imperative that the location of the tissue sample in the deep well block and the corresponding sperm sample are properly documented. Working equipment should be prepared 30 min in advance to allow for tubes to cool down. A workstation set up for cryopreservation is depicted in Fig. 2a.

Firstly a solution of 10% DMA in BSMIS (75 mM NaCl, 70 mM KCl, 2 mM CaCl_2_, 1 mM MgSO_4_, 20 mM Tris pH 8.0, sterile filtered and stored at 4 °C) is prepared and vortexed for 10 min. Meanwhile a styrofoam box is filled with dry ice and ∼0.5 l of ethanol is slowly added to create a dry ice/ethanol slush. Labelled 50 ml Falcon tubes are placed into the dry ice with only the caps visible. The number of tubes needed is determined by the number of sperm samples to be frozen down. As a general rule there should be enough tubes for 30 min of freezing as that is the time samples are incubated on dry ice before they are moved to LN_2_. A second styrofoam box is filled with wet ice to hold a rack for 0.5 ml Eppendorf tubes as well as a rack for cryovials. DMA/BSMIS aliquots for 2× (70 μl), 4× (145 μl) and/or 8× (290 μl) sample collection should be prepared in 0.5 ml tubes and labelled accordingly. Labelled cryovials are placed into an icebox so that they are chilled before use. A third styrofoam box is filled with 3 cm of LN_2_ and labelled cryoboxes (Corning Product #431121) are placed in the LN_2_ to receive the cryovials.

Males are anaesthetised (0.02% tricaine in system water) until gill movement ceases and excess water is removed by placing them on paper towels. The males should not be patted dry to avoid loss of sperm. Males are transferred onto the sponge holder (Fig. 2b) and the urogenital area is dried with a cotton bud. Sperm is expelled by gentle pressure in an anterior to posterior motion (Fig. 2c). Ideally this is done with thumb and index finger, but alternatively blunt forceps can be used. The sperm is collected into a capillary (arrow in Fig. 2d) and quantity in microlitre and quality are recorded. Quality is assessed by eye based on the milkiness of the sample and a ‘+’ is given for a very dense sample and ‘w’ given for a dilute sample, for example 2 μl of milky sperm are of quality 2+. Depending on the quantity and quality the sperm is divided into 2–8 aliquots. Samples of less than 1.5+ should not be split into more than four aliquots. At this point the quantity and quality are recorded and an appropriate number of cryovials and Falcon tubes opened ready for aliquoting. After gently expelling the sperm (avoiding bubbles) into the DMA/BSMIS (Fig. 2e) it is briefly mixed by pipetting up and down twice and quickly aliquoted into the cryovials which are immediately closed and moved into the Falcon tubes (Fig. 2f). From the point when the sperm is expelled into the cryoprotectant it should not take more than 30 s to move the samples onto dry ice as the cryoprotectant is cytotoxic. The Falcon tubes are closed immediately, hammered into the dry ice and a timer is started with the first sample such that the timepoints of samples transferred into dry ice and later to liquid nitrogen can be recorded for this and each subsequent sample.

After 30 min on dry ice samples are removed from Falcon tubes and quickly transferred into the cryoboxes in LN_2_. For permanent storage cryoboxes are transferred into liquid phase in a cryo store (Statebourne, Biosystem 12).

## 3 *In vitro* fertilisation

For *in vitro* fertilisation (IVF) females should be between 6 and 15 months old. The females should be kept together with males, well fed and on a schedule of squeezing alternated with several rounds of natural matings to synchronise the fish and ensure optimal egg production. The evening before IVF, females are set up in mating tanks with males separated by a divider. Depending on the quality of females at least six females should be allocated for each planned IVF. A single person can do the IVF procedure, but for higher throughput it is advisable to have a second person that can time incubations, record data and open cryovials.

**Fig. 3:**
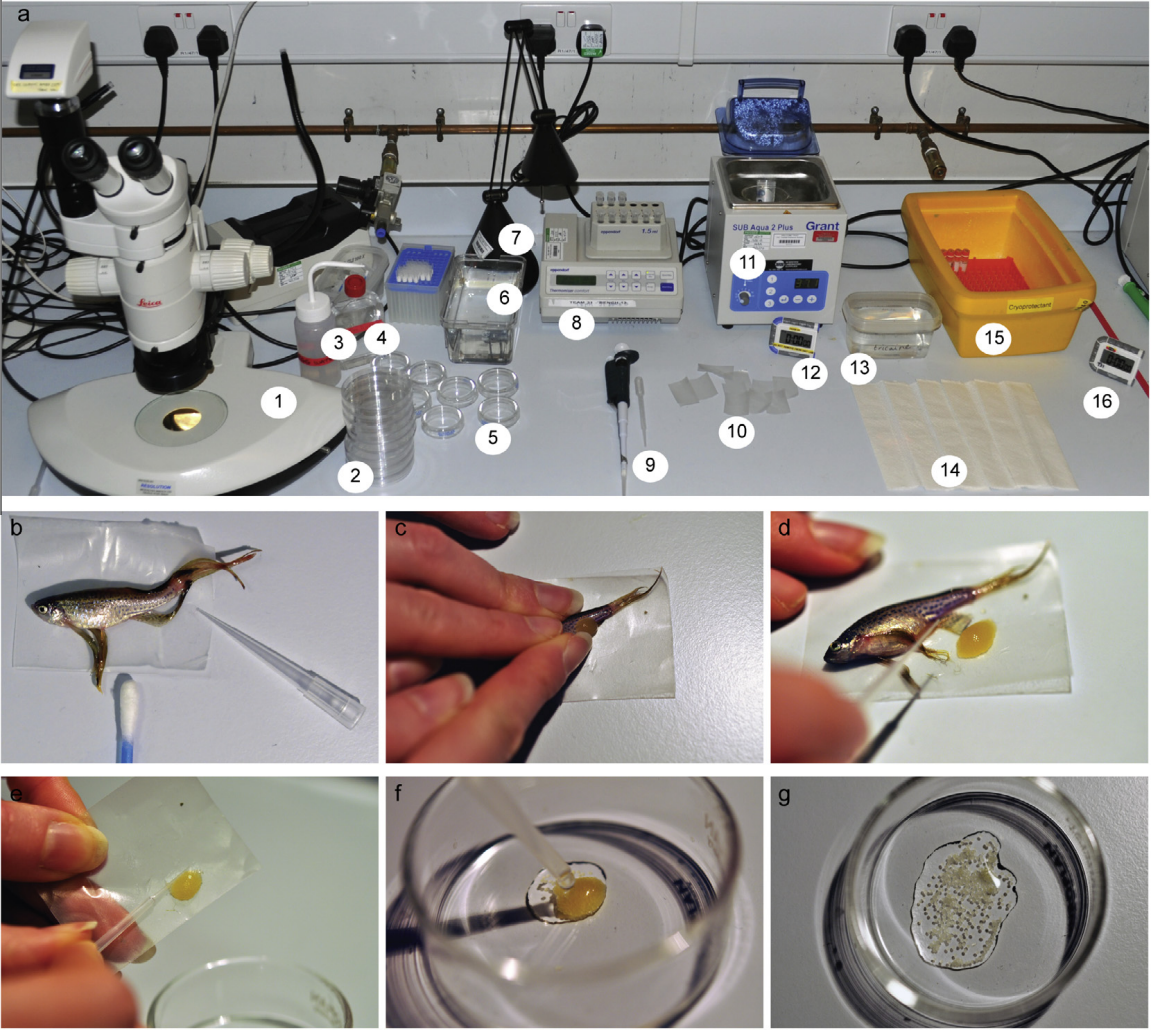
*In vitro* fertilisation. The IVF station consists of (1) a LM80 dissecting microscope, (2) 9 cm Petri dishes, egg water without Methylene Blue in a (3) squirt bottle and (4) cell culture flask, (5) 6 cm glass dishes, (6) recovery tank for females, (7) lamp, (8) heating block at 28 °C for fructose aliquots, (9) 1 ml pipette with tips and disposable 2 ml pipette, (10) pieces of Parafilm, (11) water bath at 37 °C for BSMIS, (12) and (16) timer, (13) tricaine to anaesthetise females, (14) tissues to dry females, (15) box with LN_2_ and samples to use for IVF. (a) Anaesthetised females are transferred onto Parafilm where fins are moved out of the way using a pipette tip and urogenital opening is dried with a cotton bud. (b) Gentle abdominal pressure releases eggs. (c) Eggs are separated from the female using a pipette tip (d) and transferred into glass dish. (e) Activated sperm is pipetted directly onto eggs so that they are evenly dispersed (f, g).

On the day a 50 ml Falcon tube containing BSMIS (without DMA!) is placed in a water bath at 37 °C. Aliquots of 500 μl of fructose solution (0.5% fructose and 0.018% sea salt w/v in dH_2_O) in 2 ml microcentrifuge tubes are kept in a heating block at 28 °C. A cryobox containing the sperm samples is placed in 3 cm of LN2 in an appropriate container. Fig. 3 a shows the bench set up for IVF.

Female fish are anaesthetised until gill movement stops and carefully dried on paper towels as any water left on the fish will activate the eggs and reduce overall fertilisation rates. The female is transferred onto a piece of Parafilm, the fins moved out of the way using a pipette tip and the urogenital opening dried with a cotton bud (Fig. 3b). Females are squeezed by gently applying pressure on the abdomen in an anterior to posterior motion (Fig. 3c). The eggs should be released very easily. If this is not the case the female is placed back into the tank and allowed to mate naturally. When eggs are obtained by squeezing they are separated from the female with a pipette tip (Fig. 3d) and transferred from the Parafilm into a 6 cm glass dish (Fig. 3e). The female is placed into a recovery tank with system water. A clutch suitable for IVF is yellow, translucent and holds together well, whereas watery eggs mixed with white specks should be discarded. Clutches can be pooled, but should be used within a minute to ensure that eggs do not dry out. Once a clutch of about 300–400 eggs is obtained, a cryovial is removed from the box, quickly opened and turned over to tip out any LN_2_. The tube is briefly warmed in the hand while 500 μl of 37 °C BSMIS is added. BSMIS is pipetted up and down 1–2× and stirred just until the pellet has thawed. The volume of 500 μl is pipetted immediately into 500 μl 28 °C fructose to pre-activate sperm and a disposable plastic pipette is used to immediately transfer the activated sperm onto the eggs (Fig. 3f). This addition of the sperm to the eggs should disperse the eggs evenly (Fig. 3g). Thawing and addition of activated sperm to eggs should not take longer than 40 s in total. Pre-activation of sperm increases fertility rates as sperm needs a couple seconds before it starts moving while eggs swell up immediately.

Fertilisation is allowed to take place for 1 min. Residual sperm motility can be checked after 40 s, but the dish should not be moved before that. The fertilisation time of 1 min should not be exceeded as the high salt content of BSMIS is damaging to the eggs. The dish is carefully filled with egg water (0.018% sea salt w/v in dH_2_O) without Methylene Blue (Sigma) using a cell culture flask (not a squirt bottle). After a few minutes eggs are transferred into a plastic Petri dish and topped up with more egg water. Eggs are incubated at 28 °C for at least three hours before fertility rates are measured.

When the sperm is used fresh (without freezing) for a direct IVF fertilisation rates of up to 100% are seen, however the fertilisation rate of a frozen sperm sample is lower and depends on the quality and dilution. A concentrated frozen sperm sample (e.g. one of two aliquots derived from a 2+ sample) can yield up to 50% fertilised eggs, whereas a diluted sample (e.g. one of eight aliquots derived from a 1+ sample) might only produce a 10% fertilisation rate. This can be compensated for, however, by using pools of large clutches. Complete failure of fertilisation is extremely rare.

## 4 Multi-allelic phenotyping

Due to the high mutagenic load the clutches from F2 incrosses in any ENU screen contain multiple and partially overlapping phenotypes. This is managed in a phenotype driven forward genetics screen by firstly outcrossing and thus “cleaning” phenotypes of interest through several generations. Eventually, the underlying causal mutation is identified by positional cloning. By contrast, in the ZMP approach, each phenotype is equally important and it needs to be established which of the identified mutations is causing which phenotype. For example, if incrosses of an F2 family carrying fifteen potentially disruptive mutations show three different phenotypes, a total of 45 genotype-phenotype correlations need to be tested. This does not even take into account combinatorial phenotypes that complicate the identification and isolation of a sufficient number of embryos showing the basic recessive phenotypes.

To deal with this challenge we have established a two-step triage system that takes advantage of the fact that the majority of alleles do not cause a phenotype during the first five days of development. The first round of phenotyping is comparatively quick and simple, as only morphologically normal embryos from F2 incrosses are collected and genotyped for alleles heterozygous in both parents. Alleles that do not affect the first five days of development are expected to be homozygous in 25% of the phenotypically wild-type embryos. Typically about 90–95% of alleles fall into that category and their phenotypic analysis is thus completed during the first round. Crucially, alleles that do cause a phenotype are flagged by the absence of homozygous embryos and a 1:2 ratio of homozygous wild-type to heterozygous embryos. On average about two alleles are highlighted in that manner per family.

In the second round of phenotyping F2 parents carrying the alleles flagged as phenotypic are incrossed and the resulting clutches are carefully examined. Importantly, the clutches for each of the interrogated alleles are expected to show one common phenotype. Phenotypic and non-phenotypic embryos are collected and genotyped to identify and correlate the correct phenotype.

In summary, while two rounds of phenotyping may seem counterintuitive, this approach greatly simplifies and speeds up the correlation of multiple phenotypes with multiple alleles. The first round is very quick and identifies which alleles need to be examined more closely in the second round.

### 4.1 Husbandry

Following IVF 100–150 fry are put into the nursery and raised over the next four months. Typically, about 85% of the F2 fry will survive to adulthood if raised under the same conditions as those used at ZIRC (http://www.zebrafish.org). At 3–4 months their sexes can be easily determined and the fish are sorted and reduced down to 24 males and 24 females. The fish are raised at least 1–2 months more, allowing them to increase in size and thus helping to increase clutch sizes for future crosses. The optimal time to begin crossing fish for multi-allelic phenotyping is about 6–8 months of age.

### 4.2 First round phenotyping of F2 sibling incrosses

**Fig. 4:**
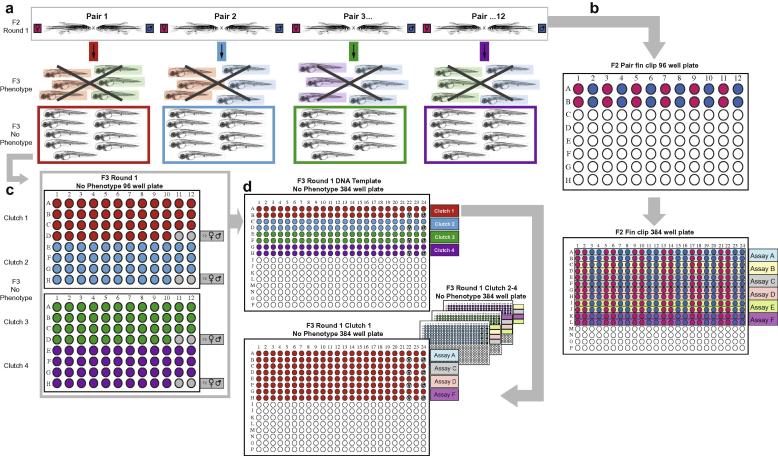
Multi-allelic phenotyping – round 1. F2 families are randomly in-crossed and the 12 largest clutches are collected and enter the pipeline with their respective parents. 150–200 fertilized F3 embryos are raised till 5 dpf, all abnormal fry are removed and 46+ phenotypically normal fry are collected and frozen in MeOH. (a) Fin clips are taken from each F2 parent and placed into a 96 well plate. Genomic DNA is prepared from the tissue and transferred into 384 well plates allowing for up to 24 KASP™ genotyping assays per 384 well plates, with all disruptive mutations identified in the original F1 male being interrogated. (b) The 46 phenotypically normal embryos from each clutch are aliquoted into half of a 96 well plate, DNA is extracted, and aliquots of the corresponding parental DNA are added to the last two wells. (c) The 96 well plates are transferred into 384 well plates and each clutch is stamped out into an individual 384 well plate and assayed for all alleles that both parents were heterozygous for (d).

All F2 siblings are randomly incrossed as individual pairs (Fig 4a). Embryos are collected from the 12 largest clutches and fin-clips for DNA preparation are taken from their F2 parents. For fin-clipping the fish are anaesthetised in 0.02% tricaine, transferred with a tea strainer onto a Petri dish lid and 3–4 mm from the tip of the tail fin are amputated with a scalpel. The fin clips are transferred to individual wells of a 96-well Abgene SuperPlate™ using forceps. The fin-clip from the pair one female goes in well A1, the male in A2 etc. The pairs are transferred into holding tanks which are labelled according to the position of their fin clip in the 96-well plates, so that the female from the first pair is A1 and the male A2, the female from pair 2 is A3 and the male from pair 2 A4 etc. (Fig. 4b).

From each clutch 150–200 fertilised embryos are sorted into 3–4 9 cm Petri dishes containing egg water (1 l H_2_O, 0.18 g sea salt, 500 μl 2 mg/ml Methylene Blue) with 50 embryos per dish. Each dish is labelled with the family number, the parental IDs and the date. These dishes are kept in a 28 °C incubator and checked daily to remove any dead or obviously phenotypic embryos.

On 5 dpf, 50 phenotypically wild-type fry are collected from each clutch (Fig. 4a). To assess which fry have no observable phenotype, the dish is placed under a Leica M80 dissecting microscope using a 1.0× objective, with halogen lighting from below and an LED lighting ring from above. A simple behavioural and morphological analysis is performed. The swimming behaviour of the fry is assayed by touching the tail with forceps, and a startle reflex is assayed by tapping the side of the dish with forceps. Any fry exhibiting abnormal behaviour are discarded. Fry are then anaesthetised by adding approximately 1 ml of 0.4% tricaine to the dish. Using a higher magnification on the Leica M80 the melanocytes, iridophores and xanthophores, the digestive organs (liver, intestine and pancreas), the swimbladder, muscle, eyes, fins, jaw, ear, heart and circulation are evaluated. Table 1 shows which aspects of development are assessed. Any fry with abnormal morphology are discarded, leaving just those with no observable behavioural or morphological phenotype abnormalities. Using a Pasteur pipette the phenotypically wild-type fry are transferred into a 1.5 ml microcentrifuge tube, labelled with the family stock number and parent IDs, the egg water is replaced with 1 ml of 100% methanol to fix the embryos and the embryos are stored at −20 °C. Some fry may stick to the walls of the Pasteur pipette, so it is important to check the pipette regularly.

#### 4.2.1 Genotyping of adult fin clips and F3 fry

##### 4.2.1.1 Extracting DNA

Each phenotypically wild-type fry is transferred into an individual well of a 96-well Abgene SuperPlate™ using forceps in a fume hood, so that fry from clutch A1A2 fill wells A1 to D10, fry from clutch A3A4 fill wells E1 to H10 of plate 1, fry from clutch A5A6 fill wells A1-D10 of plate 2 etc. (Fig. 4 c). At this stage wells D11, D12, H11 and H12 are left empty. The plates are left at room temperature until the methanol has evaporated. Alternatively, plates can be placed on a heating block to speed up the evaporation. To extract DNA from the fry, 25 μl of HotShot[24] base solution (freshly prepared from 50× stock solution: 1.25 M NaOH, 0.01 M EDTA) is added to each well using a multidrop (Thermo Multidrop Combi). The plates are heat sealed and heated to 95 °C for 30 min on a PCR block with heated lid. The plates are then vortexed and 25 μl of HotShot neutralisation buffer (freshly prepared from 50x stock solution: 2 M TRIS–HCl) is added to each well using a multidrop. The plates are resealed and vortexed, and the DNA can be stored at −20 °C or used straight away for genotyping. Fin clips are processed in the same way as embryos but 50 μl each of HotShot base solution and neutralisation solution are used.

##### 4.2.1.2 KASP™ genotyping

Genotyping is performed using KBioscience’s Competitive Allele-Specific PCR SNP genotyping system (KASP™) [19] in a 96 or 384 well plate format. KASP™ uses fluorescent allele-specific forward primers with a common reverse primer to amplify genomic DNA. Individual assays specific for each detected mutation are designed by submitting a 50 bp region around the mutation to KBioscience along with any other known SNPs or insertions/deletions so that they can be avoided.

The plates are read using a suitable plate reader such as a PHERAstar plus (BMG labtech) and the software KlusterCaller (KBioscience), and the genotype of each sample scored as homozygous wild-type, heterozygous or homozygous mutant based on clustering of the samples. The genotypes are visually represented such that each sample is a spot on a plot, with homozygous mutants forming a cluster of spots in the top left corner, heterozygous samples form a cluster of spots near the centre of the graph, and wild-types form a cluster at the bottom right (Fig. 6d). A minimum of 24 samples should be used to ensure efficient clustering of genotypes in each assay.

##### 4.2.1.3 Genotyping F2 fin clips

Pipetting by hand from a 96 well plate into a 384 well plate and from one 384 well plate into another is tedious and prone to errors, therefore it is recommended that a liquid handling robotic system such as the Agilent Bravo™ be used when undertaking high-throughput genotyping. F2 fin clips are genotyped for all mutations present in the founder. The F2 DNA plate is defrosted if frozen, and 30 μl pipetted into a 384 deep well plate (Greiner bio-one). If there are more than 6 and up to 12 pairs, each DNA sample is pipetted in duplicate so that the DNA for female A1 is now in wells A1 and A2, and the DNA from male A2 is now in well A3 and A4 etc., filling a maximum of two rows per genotyping assay thus providing a sufficient number of samples for the genotyping assay (Fig. 4b). If there are fewer than six pairs then each DNA is pipetted in quadruplicate such that each assay again occupies two rows. A volume of 90 μl of fresh dH_2_O is added to each well to dilute the DNA using the multidrop. A volume of 2 μl of the diluted DNA is aliquoted into twin.tec PCR 384 well plates (Eppendorf). Eight assays (2 rows per assay) can be run on a single plate, so the number of plates required will vary depending on the number of assays for that particular family. The aliquoted DNA is dried by leaving plates on a 65 °C heat block for 45 min.

While the DNA is drying, KASP™ reaction mixes are set up. A single reaction contains 2 μl 2× KASP™ reagent, 0.055 μl assay mix, 0.032 μl MgCl_2_ and 1.913 μl dH_2_O. So for each assay enough reaction mix is made for 50+ reactions, which ensures there is enough volume for the 48 required reactions (2 rows on the plate). If many assays are needed, a mastermix excluding the different assay primers can be prepared. A volume of 4 μl of reaction mix is added to the DNA, with up to 8 different assays per plate. The plates are heat sealed and run on a PCR machine with the following program: (1) 94 °C 15 min, (2) 94 °C 10 s, (3) 57 °C 5 s, (4) 72 °C 10 s, repeat steps (2)–(4) for 21 cycles, (5) 94 °C 10 s, (6) 57 °C 20 s, (7) 72 °C 40 s, repeat steps (5)–(7) for 19 cycles, hold at 10 °C.

**Fig. 5:**
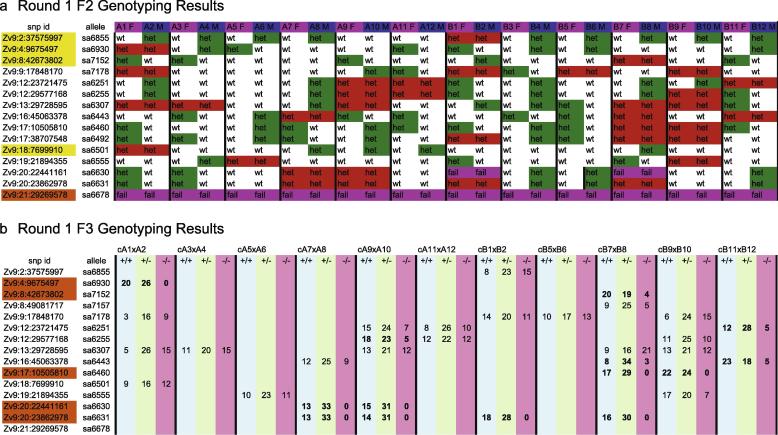
Genotyping results. An example of first round genotyping results from F2 parents and their non-phenotypic F3 progeny. The genomic position of each mutation is in the column labelled *snp id* followed by the allele designation directly to the right. The top row labels first the female of the pair followed by the male. Their IDs stem from their original positions in a 96 well plates. In total 12 pairs are shown. Red boxes indicate alleles where both parents are carriers, whereas green signifies alleles with only one heterozygous parent in a pair. The *snp id* which are highlighted in yellow indicate alleles where only one cross contains heterozygous parents. The orange highlighted *snp id* flags assays which require attention. In the case of *sa6678* all samples have failed, which indicates that this is probably due to the assay not working either at the amplification or clustering step. (a) For each clutch, genotyping is carried out on 46 non-phenotypic 5 dpf fry for all alleles that both F2 parents are heterozygous for. Absolute numbers of genotypes per allele and clutch are shown. The alleles flagged in orange in the *snp id* column indicate alleles suspected of causing a phenotype and should be considered for second round phenotyping (b).

Plates containing poorly grouped assays are resealed and put back on the PCR machine for an extra 10 extension cycles (PCR program (1) 94 °C 10 s, (2) 94 °C 10 s, (3) 57 °C 20 s, (4) 72 °C 40 s, repeat steps (2)–(4) for 10 cycles, hold at 10 °C). Fig. 5a shows an example of how the F2 genotype data can be recorded.

##### 4.2.1.4 Genotyping of first round non-phenotypic embryos

Round one fry from each clutch are genotyped for alleles that are heterozygous in both parents. Round one fry DNA plates are defrosted if frozen, and 10 μl of parental DNA from the F2 plate is added into the empty wells as controls, so that for clutch A1A2, DNA from the mother A1 is added to D11 and DNA from the father A2 is added to D12 and for clutch A3A4 DNA from the mother A3 is added to H11 and DNA from the father A4 is added to H12 etc. DNA is transferred from each 96 well plate into a 384 deep well plate (Costar, VWR) (Fig. 4d) and diluted 1 in 4 with dH_2_O. The liquid handling robotic system pipettes using a 12-tip pipetting head, such that rows A and B on a 96 well plate intercalate in row A on a 384 well plate. Volumes of 2 μl of diluted DNA for each clutch are aliquoted into a further twin.tec PCR 384 well plate (Eppendorf) in replicates according to the number of assays to be performed. The DNA is dried and the reaction mixes made up as before. A volume of 4 μl of reaction mix is added to the DNA and up to eight assays can be performed on each plate. The number of plates needed for each clutch will vary depending on how many alleles were heterozygous in both parents. The plates are heat-sealed and the same PCR program used as for the fin clips. Plates are read in the same way as for fin clips and extra amplification cycles added when necessary. An example of F3 round 1 genotype data is given in Fig. 5b.

### 4.3 Second round phenotyping of F2 sibling incrosses

Round one genotyping results are analysed using a Chi-squared test (*p*-value cut-off <0.05) to flag alleles for which homozygosity is reduced in the wild-type fry (Fig. 5b). Mutations found to be homozygous in the phenotypically wild-type fry at greater than the cut-off value are determined not to cause an observable phenotype during the first five days of development. Any mutations that are homozygous at a frequency below the cut off value are suspected to cause a phenotype and are carried forward to the second round of phenotyping.

**Fig. 6:**
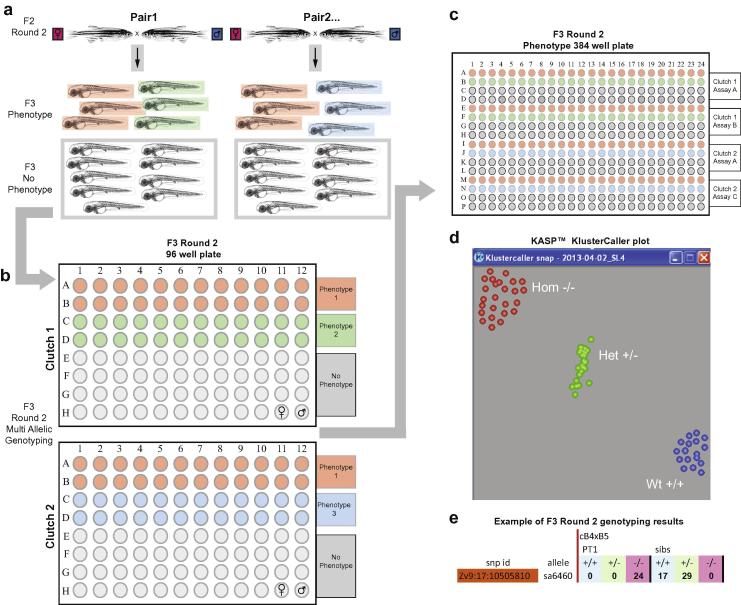
Multi-allelic phenotyping – round 2. Round 2 pairs are set up based on the genotyping results of the first round phenotyping. Clutches are collected and observed over the first five days of development. (a) When phenotypes arise in approximately 25% of embryos, they are collected along with non-phenotypic siblings and frozen in MeOH. Multiple phenotypes per clutch can be collected simultaneously but are archived in separate tubes. Phenotypic embryos are then aliquoted into the upper half of a 96 well plate. (b) Multiple phenotypes can be added to a single plate and multiple plates per clutch can be produced. Phenotypically normal siblings are then added to the lower half of the 96 well plates with wells H11 and H12 containing parental control DNA. The 96 well plates are then transferred into 384 well plates where all alleles flagged for each particular pair combination are assayed against all identified phenotypes per clutch. (c) A KASP Kluster Caller plot depicting the grouping of the three genotypes. (d) This is an example of a genotype-phenotype association that would be documented as likely causal (e).

In the second round of phenotyping, F2 carriers of the allele of interest are re-crossed (Fig. 6a), and embryos from successful matings collected and labelled with the family stock number and parent IDs. Fertilised embryos are sorted into 9 cm Petri dishes containing egg water, 50 embryos per dish, keeping as many embryos as possible for each clutch. The number of embryos kept per clutch is documented. Dishes are kept in a 28 °C incubator and checked daily. Any dead embryos are removed and the number documented. The clutches are examined on each of the first five days of development and as soon as a phenotype is visible, the phenotypic fry are collected, photographed using a Leica DFC 450 CCD camera and fixed in methanol (Table 1). Each phenotype is given a unique ID such as “*family-stock-number*”_A1A6_1; A1 and A6 are the parents, and ‘1’ indicates that this was the first phenotype observed in this clutch. On 5 dpf, up to 46 phenotypically wild-type fry are also collected.

For each clutch all phenotypic and non-phenotypic fry are genotyped for the suspected causative allele. For each clutch, fry of each phenotype are placed into individual wells of a 96-well plate, and the remainder of the wells is filled with individual non-phenotypic fry, excluding H11 and H12, which are left empty for parental control DNA (Fig. 6b). The content of each well is recorded. DNA is extracted using HotShot solutions and F2 parental DNA is added to wells H11 and H12. The DNA is diluted 1:4 by adding dH_2_O to each well using the multidrop. The diluted DNA is replicated into a 384 well plate. A single clutch can be aliquoted up to 4 times on a plate, which means that 4 assays can be performed per plate (Fig. 6c). The DNA is dried and enough KASP™ mixes made up for 96 reactions (4 rows on the 384 well plate). A volume of 4 μl of reaction mix is added to each well, the plates are heat-sealed and the same KASP™ PCR program used as before. When multiple suspected causative alleles are present in the same clutch, the plate is genotyped for all of those alleles.

When all the fry with a particular phenotype are homozygous for the allele of interest, and all the non-phenotypic fry are heterozygous or wild-type, then the allele is documented as being likely to cause that phenotype (Fig. 6e). The photographs are then uploaded onto the ZMP website along with a description of the phenotype.

## 5 Phenotype annotation

Phenotype documentation consists of live images acquired on a Leica M80 along with text annotation. For downstream data mining and analysis, phenotypic annotations need to be searchable by various terms such as tissue type, anatomical entity or developmental stage. This is best achieved by adhering to controlled sets of defining terms that are species independent where possible. Catalogues of these terms are grouped into ontologies, where terms of increasing detail are structured in a tree relationship from root (little detail) to leaf (greatest detail). The ontologies used for zebrafish phenotype annotation are listed in Table 2 and can be browsed at the Ontology Lookup Service OLS (http://www.ebi.ac.uk/ontology-lookup/init.do).

Phenotypic annotations follow an entity-quality (E-Q) syntax. The entity can be an anatomical or cellular structure, spatial reference, molecular or cellular function as well as a biological or molecular process. Entities are broadly split into continuants (anatomical, cellular or molecular components) and occurrents (molecular or biological processes). Different entity terms can be combined (“post-composition”) as super- and subterms to describe phenotypes with greater specificity, however only certain combinations of entities are allowed. For example, anatomical ontology (AO) terms can be both super- and subterms, whereas a gene ontology (GO) molecular function (e.g. phosphorylase activity) can only be used as a subterm and GO biological processes cannot be post-composed at all. Finally, relational qualities between two independent entities, i.e. terms that are not in a child-parent relationship, can be described in an entity-quality-entity syntax.

**Fig. 7:**
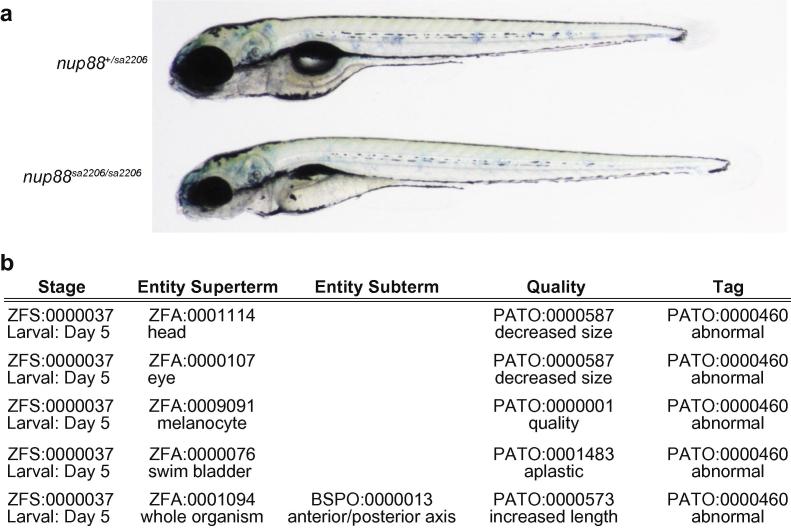
An example of phenotype annotation. At 5 dpf *nup88^sa2206^* mutants have smaller heads and eyes, abnormal melanocytes, increased body length and lack the swim bladder. (a) Entity quality annotations for *nup88^sa2206^* phenotype (b).

A phenotype will normally need multiple E-Q annotations to be described fully. In addition, each annotation gets a tag of abnormal or normal. Most annotations will have the tag “abnormal”, however, where a “normal” phenotype is noteworthy it should be annotated. For example, if melanocytes and iridophores are reduced in numbers, but xanthophores are unaffected, the xanthophore phenotype should be annotated and tagged as normal while the melanocyte and iridophore phenotypes would be tagged as abnormal. An annotation example is given in Fig. 7. For more in depth information on phenotype annotation, please contact www.zfin.org.

## 6 Concluding remarks

The methods presented here allow for the rapid and simultaneous analysis of multiple phenotypes and their linkage to causative mutations. While the described pipeline focuses on early embryonic phenotypes in live fry, the approach can be extended to assays involving live or fixed stainings, molecular analysis and stages beyond embryogenesis. The combination of systematic phenotyping with simultaneous genotyping blurs the border between the two conventional genetics approaches as data on phenotype-genotype associations (“reverse genetics”) as well as unlinked phenotypes (“forward genetics”) can be recorded. Currently, this project focuses on stop and essential splice-site mutations within the annotated coding regions of the zebrafish genome. Phenotypes caused by other mutations are observed, but currently not followed up further. It is conceivable, however, to combine this effort with a simultaneous forward genetics screen, particularly in conjunction with new sequence based mutation mapping methods [25–27].

Crucially, archives of cryopreserved sperm samples in multiple aliquots together with their corresponding genomic DNA samples create the opportunity to re-analyse the DNA for any targets that might become interesting in the future, such as non-coding regulatory elements or other genomic features yet to be discovered.

Bespoke targeted genome manipulation [28,29] will complement random mutagenesis to achieve the ultimate goal of complete functional annotation of the zebrafish genome.

## Author information

Zebrafish were maintained in accordance with UK Home Office regulations, UK Animals Scientific Procedures Act 1986 under the project licence authority. This licence was reviewed by The Wellcome Trust Sanger Institute Ethical Review Committee. The authors declare no competing financial interests. Correspondence should be addressed to E.B.N. (emb@sanger.ac.uk).

## Figures and Tables

**Table 1:** Schedule for phenotyping. Each round of phenotyping occurs over 6 days. During round 1 phenotyping, dead or abnormal embryos are removed on each day, and on 5 dpf non-phenotypic embryos are collected, which requires checking that all aspects of development are normal. During round 2 phenotyping, dead embryos are discarded but aspects of development are checked on each of the first 5 dpf. Phenotypic embryos are collected and photographed as soon as the phenotype is apparent, and on 5 dpf 46 non-phenotypic embryos are collected.

**Table 2:** Ontologies used in zebrafish phenotype annotation. A summary showing the ontological terms used in describing zebrafish phenotypes.

Ontology name	Abbreviation	Covered terms	Syntax component
Zebrafish anatomy and development	ZFA/ZFS	Anatomical entities and developmental stages	Entity
Gene ontology	GO	Cellular components. Cellular and molecular functions. Molecular and biological processes.	Entity
Spatial reference ontology	BSPO	Anatomical regions	Entity
Phenotype and trait ontology	PATO	Phenotypic qualities	Quality
